# Medial repair band: independent risk factor for collapse in traumatic osteonecrosis of the femoral head

**DOI:** 10.3389/fmed.2026.2001241

**Published:** 2026-10-26

**Authors:** Yanyu Wang, Changyu Huang, Zhibing Gong, Ziqi Li, Wei He, Zhaoke Wu, Zhikun Zhuang

**Affiliations:** 1 Quanzhou Orthopedic Clinical Medical College, Fujian University of Traditional Chinese Medicine, Quanzhou, Fujian, China; 2 Department of Orthopaedic Surgery, Quanzhou Orthopedic-Traumatological Hospital, Quanzhou, Fujian, China; 3 Department of Orthopaedic, The Third Affiliated Hospital of Guangzhou University of Chinese Medicine, Guangzhou, China

**Keywords:** femoral head collapse, femoral neck fracture, magnetic resonance imaging, repair band, traumatic osteonecrosis of the femoral head

## Abstract

**Objective:**

Femoral head collapse is closely associated with a poor prognosis in osteonecrosis of the femoral head (ONFH). This study aimed to evaluate the association between the MRI repair band and the progression to collapse in traumatic osteonecrosis of the femoral head (TONFH).

**Methods:**

We retrospectively analyzed TONFH patients admitted from January 2016 to May 2023. The following clinical data were collected: patient age, sex, affected side, presence of complete fracture displacement, performance of emergency surgery, achievement of anatomical reduction, and other relevant clinical characteristics. Median follow-up was 44.5 months, with collapse >2 mm as the survival endpoint. Univariate and multivariate regression analyses assessed the association between MRI medial repair band and collapse. Inter-observer consistency was evaluated using Kappa test. We also analyzed the association between fracture displacement and MRI medial repair band.

**Results:**

148 TONFH patients were included [median age 46.50 years (IQR: 38.50–52.00); 67(45.27%) males, 81(54.73%) females]. Collapse occurred in 63 (collapse group) vs. 85 (non-collapse group). The interobserver reliability of the medial repair zone, as measured by the intraclass correlation coefficient (ICC), was 0.802.Multivariate analysis identified the medial repair band as an independent risk factor for collapse (OR: 13.350, 95% CI: 5.767–30.907; *P* < 0.001). Survival was significantly lower with a medial vs. lateral repair band (*P* < 0.001). Garden type IV fracture was significantly associated with medial repair band formation (OR: 2.581, 95% CI: 1.304–5.106; *P* = 0.006).

**Conclusion:**

The medial repair band on MRI is an independent risk factor for collapse in patients with TONFH; furthermore, Garden type IV is closely associated with the formation of the medial repair band. The MRI medial repair band is an imaging feature for assessing the risk of collapse in TONFH.

## Introduction

Traumatic osteonecrosis of the femoral head (TONFH) is a common and severe complication following femoral neck fracture (FNF) (1, 2). The pathogenesis of TONFH is closely associated with inadequate blood supply to the femoral head (3). Femoral head collapse caused by avascular necrosis leads to the loss of hip joint function (4). Early identification of the risk of collapse and subsequent intervention can provide a therapeutic window to delay or even avoid total hip arthroplasty (5).

The ARCO staging system (Association Research Circulation Osseous) (6), which is based on the assessment of the degree of femoral head collapse, and the JIC classification (Japanese Investigation Committee) (7), which evaluates the ONFH lesion area, are widely used to guide the treatment of ONFH. They are considered effective staging and classification schemes. However, both ARCO staging and JIC classification are assessments performed after ONFH has already occurred. For patients with femoral neck fractures, both the patients themselves and clinicians desire the ability to predict the occurrence of TONFH before it happens, as this would alter treatment decisions. MRI is recognized as the gold standard for diagnosing ONFH, and this holds true for TONFH as well. Kawasaki et al. (8) confirmed that early MRI imaging following cannulated screw fixation for FNF can effectively indicate the presence of ONFH. Furthermore, with iterations in the metal composition of cannulated screws and the development of metal artifact reduction sequences (MARS) in MRI technology (9), the issue of degraded MRI quality due to metal artifacts from cannulated screws has been mitigated.

The repair band is a specific sign of ONFH on MRI, appearing as a low-signal band on T1-weighted images (T1WI) and a double-line sign on T2-weighted images (T2WI), it represents a transition zone between necrotic bone and newly formed bone (6), reflecting the body's attempt to biologically “seal” the necrotic focus through local bone remodeling. Its border is a sclerotic band formed by new bone tissue (10–12). Although the repair band can contribute to stress distribution, it may also impede blood supply from reaching the femoral head (13, 14). Concurrently, an imbalance in bone remodeling leads to the accumulation of micro-fractures, which can extend to the subchondral bone, ultimately resulting in subchondral collapse (15). This suggests a potential correlation between the presence of a repair band in TONFH and femoral head collapse. Notably, TONFH and non-traumatic ONFH exhibit significant differences in pathogenesis and repair responses (16). Non-traumatic ONFH is influenced by systemic factors, leading to necrotic lesions distributed across the medial, central, and lateral regions of the femoral head. Consequently, the location of the repair zone varies with the site of the lesion, wherein patients with involvement of the lateral weight-bearing area face a high risk of collapse (7, 17). In contrast, TONFH originates from injury to the lateral epiphyseal arteries, resulting in necrotic areas predominantly involving the lateral weight-bearing region, while isolated medial or central involvement is rare (8, 18). Correspondingly, the distribution of repair zones undergoes characteristic alterations. Therefore, investigating the specific features of repair zones in TONFH holds significant clinical importance.

Here, we hypothesize that the presence of a repair band on MRI is a risk factor indicative of collapse in TONFH.

## Materials and methods

This Single-center retrospective cohort study was conducted in accordance with the Declaration of Helsinki and approved by the Institutional Review Board of our institution (Ethics Approval No.: IRB-2023-043). All participants signed informed consent forms.

### Patient selection

Patients who underwent cannulated screw fixation for FNF at our institution between January 2016 and May 2023 were included in this study (Figure 1). The inclusion criteria were as follows: (1) traumatic FNF in patients aged 18–60 years; (2) treatment with closed reduction and internal fixation using cannulated screws. Exclusion criteria included: (1) concomitant hip diseases such as rheumatoid arthritis or gouty arthritis; (2) non-traumatic FNF, including pathological fractures (e.g., due to neoplasms); (3) incomplete clinical or imaging data. Data on patient age, sex, affected side, and imaging findings were collected from the hospital medical record system. All patients were followed for at least of 2 years.

**Figure 1:**
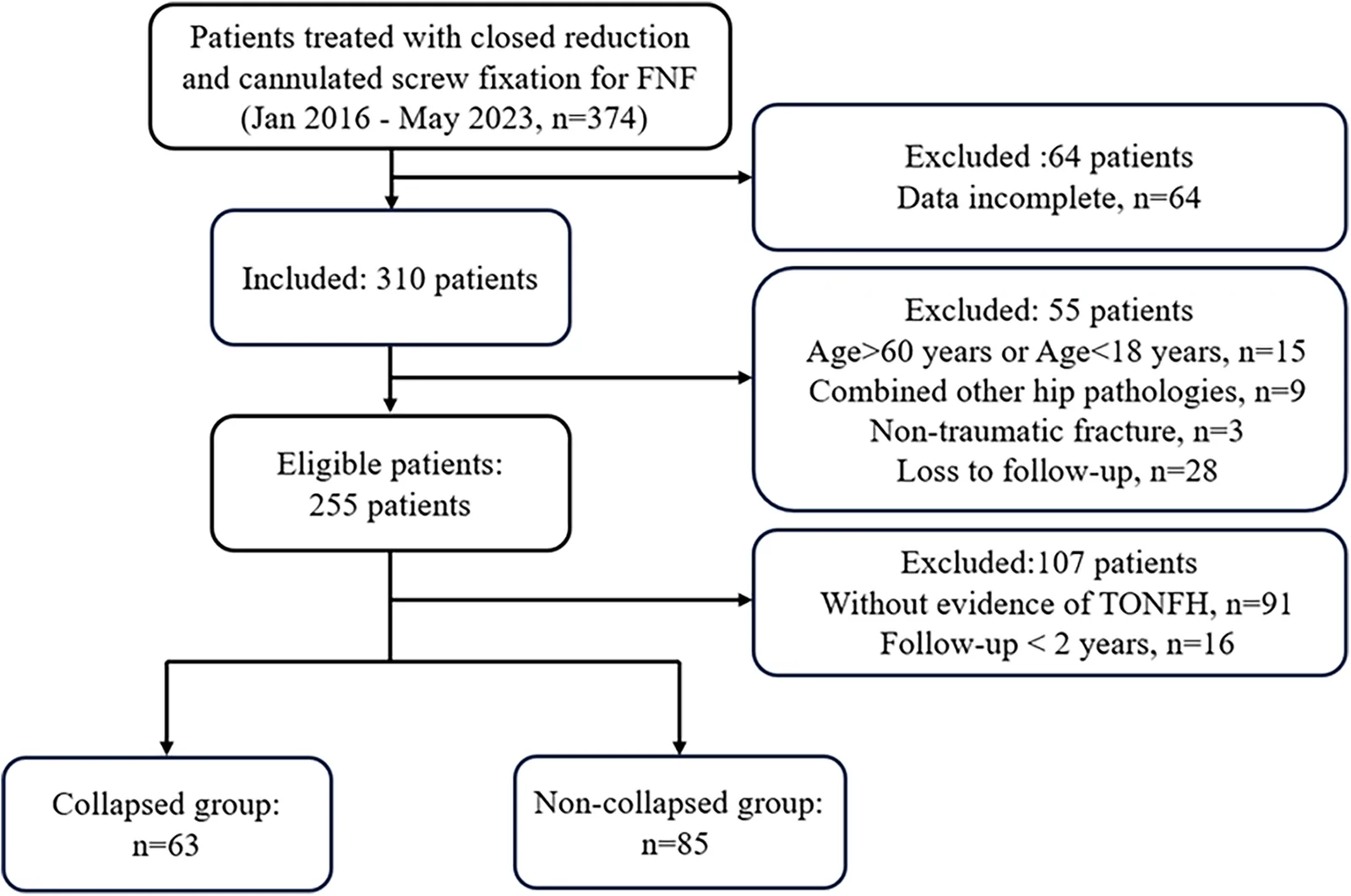
Flow diagram of the current study.

### Surgical procedure and postoperative rehabilitation

All surgeries were performed by the same surgical team. Briefly, under fluoroscopic guidance, satisfactory fracture reduction was achieved through traction, internal rotation, and abduction maneuvers. Three cannulated screws were then inserted into the femoral head in an inverted triangular configuration. Postoperatively, patients routinely received analgesia and anticoagulation therapy. Weight-bearing was initiated at 3 months postoperatively. Follow-up assessments were conducted regularly at 1, 2, 3, 6, 12, 18, and 24 months postoperatively, and annually thereafter. All patients underwent MR examination at 3 months postoperatively.

### Imaging evaluation

Standard anteroposterior pelvic and lateral femoral neck radiographs were obtained preoperatively and postoperatively. Two senior surgeons independently assessed the Garden classification and reduction index, with any disagreements resolved by consensus involving a third senior surgeon. Garden types I, II, and III were defined as non-completely displaced fractures, whereas type IV was defined as a completely displaced fracture. ONFH was staged according to the ARCO system (6). On MRI, the repair band was identified as a low-signal-intensity band on T1WI or a double-line sign on T2WI (Figure 2) (19). The points at which the repair band intersected the subchondral bone were designated as its lateral start point and medial end point (Figures 2A2). Based on coronal MRI, the repair band was classified as lateral if it did not extend to or beyond the fovea of the ligamentum teres (Figures 2A2, 3A2), and as medial if it involved or extended beyond the fovea (Figures 2B2, 3B2). Previous studies have confirmed that femoral head collapse>2 mm is significantly associated with worsening symptoms and the need for secondary surgical intervention (20, 21). In the present study, femoral head collapse >2 mm was defined as the endpoint event (Figures 2B4, 3B4), measured on radiographs by comparing sequential images with the earliest post-reduction films.

**Figure 2:**
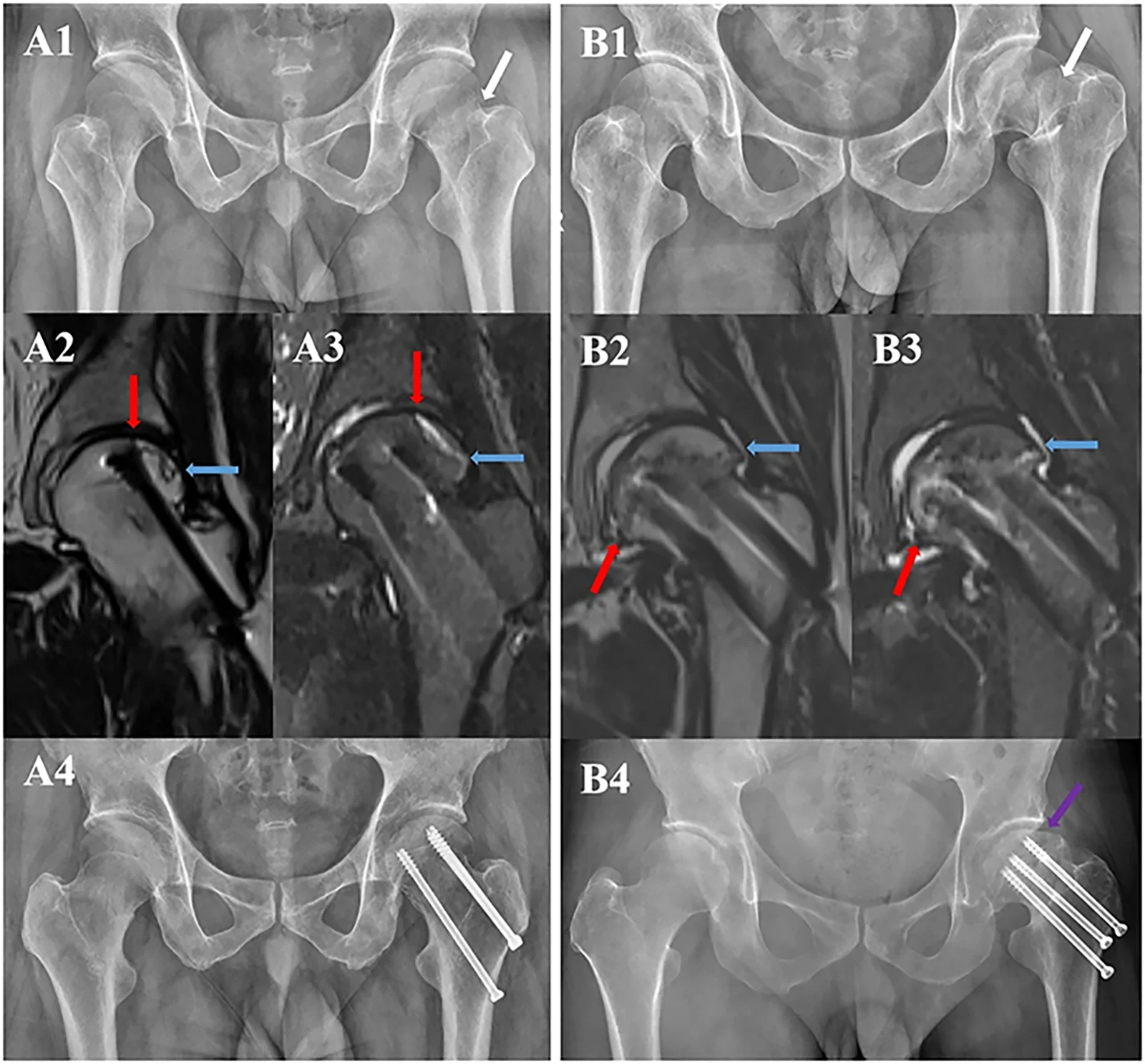
Different outcomes in two cases with distinct fracture types. The white arrow indicates the fracture site; the blue arrow indicates the starting point of the repair band; the red arrow indicates the endpoint of the repair band; the purple arrow indicates the collapsed femoral head. **(A)** Male, Garden type II fracture, exhibiting a lateral repair band with no subsequent collapse of the femoral head; **(B)** male, Garden type IV fracture, exhibiting a medial repair band with eventual collapse of the femoral head; **(A1,B1)** preoperative anteroposterior radiographs of the hip; **(A2,B2)** T1WI at 3 months postoperatively; **(A3,B3)** T2WI; **(A4)** anteroposterior radiograph of the hip at 31 months postoperatively; **(B4)** anteroposterior radiograph of the hip at 21 months postoperatively.

**Figure 3:**
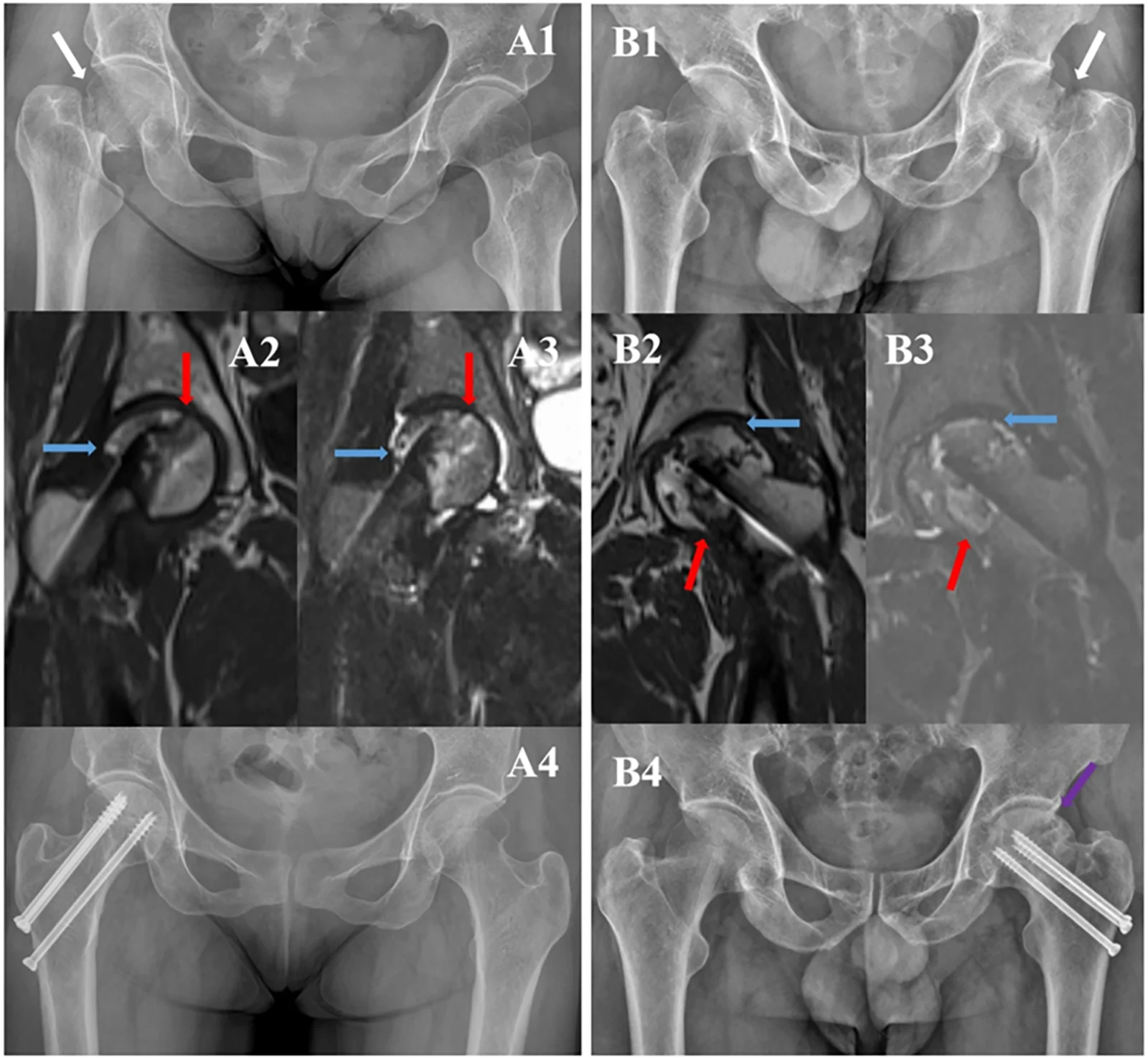
Different outcomes in two cases with completely displaced fractures. The white arrow indicates the fracture site; the blue arrow indicates the starting point of the repair band; the red arrow indicates the endpoint of the repair band; the purple arrow indicates the collapsed femoral head. **(A)** Female, Garden type IV fracture, exhibiting a lateral repair band with no subsequent collapse of the femoral head; **(B)** male, Garden type IV fracture, exhibiting a medial repair band with eventual collapse of the femoral head; **(A1,B1)** preoperative anteroposterior radiographs of the hip; **(A2,B2)** T1WI at 3 months postoperatively; **(A3,B3)** T2WI; A4. Anteroposterior radiograph of the hip at 37 months postoperatively; **(B4)** anteroposterior radiograph of the hip at 17 months postoperatively.

### Statistical analysis

Continuous variables with normal distribution are presented as mean ± standard deviation (*x¯* ± *s*), while non-normally distributed variables are described using median and interquartile range. Categorical variables are summarized as frequencies and percentages. Differences between groups for normally distributed continuous variables were compared using the Student's *t*-test, and the Mann–Whitney *U* test was used for non-normally distributed variables. Categorical data were compared using the chi-square test or Fisher's exact test, as appropriate Significant predictors identified from univariate analyses were incorporated into a multivariate logistic regression model. Results are expressed as odds ratios (OR) with corresponding 95% confidence intervals (CI). Inter-observer agreement was assessed using the Kappa statistic. All statistical analyses were performed using SPSS for Windows (Version 27.0; IBM Corp., Armonk, NY, USA). A two-sided *P*-value <0.05 was considered statistically significant. Survival curves were generated using the Kaplan–Meier method, and differences between groups were compared with the log-rank test. GraphPad Prism 9.0.0 (GraphPad Software, San Diego, CA, USA) was used to plot the survival curves.

## Results

A total of 374 patients were initially evaluated, after excluding 64 patients due to incomplete clinical or radiographic data, leaving 310 patients. An additional 55 patients were excluded based on the following pre-defined criteria: age >60 or <18 years (*n* = 15), concomitant hip diseases (*n* = 9), non-traumatic fractures (*n* = 3), and loss to follow-up (*n* = 28). Among the remaining 255 patients, a further 107 were excluded due to the absence of TONFH at the final follow-up (*n* = 91) or follow-up < 2 years (*n* = 16). Consequently, a total of 148 patients (148 hips) were included (Figure 1), with a median follow-up duration of 44.50 (IQR: 32.00–57.70) months. Among these, 63 hips were classified into the collapse group and 85 into the non-collapse group. The median age was 47.00years (IQR: 43.00–52.00) in the collapse group and 45.00 years (IQR: 38.00–52.00) in the non-collapse group. No statistically significant differences were observed between the two groups regarding age, sex distribution, affected side, achievement of anatomic reduction or performance of emergency surgery (Table 1). The interobserver intraclass correlation coefficient (ICC) for identifying the medial repair band was 0.802, indicating excellent reliability.

**Table 1:** Characteristics of included patients with TONFH.

Variable	Total	Collapse group	Non-collapse group	*P*-value
Age (years)	46.50 (38.50, 52.00)	47.00 (43.00, 52.00)	45.00 (38.00, 52.00)	0.766
Gender, *n* (%)				0.408
Male	67 (45.27)	31 (49.21)	36 (42.35)	
Female	81 (54.73)	32 (50.79)	49 (57.66)	
Laterality, *n* (%)				0.901
Left	79 (53.38)	34 (53.97)	45 (52.94)	
Right	69 (46.62)	29 (46.03)	40 (47.06)	
Complete Displacement (Garden IV), *n* (%)				0.021
Yes	68 (45.95)	36 (57.14)	32 (37.65)	
No	80 (54.05)	27 (42.86)	53 (62.35)	
Surgery within 24 h, *n* (%)				0.862
Yes	96 (64.86)	40 (63.49)	56 (65.89)	
No	52 (35.14)	23 (36.51)	29 (34.12)	
Anatomical Reduction, *n* (%)				0.135
Yes	86 (58.11)	30 (47.62)	56 (65.89)	
No	62 (41.89)	33 (36.51)	29 (34.12)	
MRI Repair Band Type, *n* (%)				<0.001
Medial	76 (51.35)	53 (84.13)	23 (27.06)	
Lateral	72 (48.65)	10 (15.87)	62 (72.94)	
Follow-time (months)	44.50 (32.00, 57.70)	45.00 (30.00, 67.00)	44.00 (32.00, 53.00)	0.526

Quantiles and *P*-values for continuous variables were calculated using the Mann–Whitney *U* test; *N* (%) and *P*-values for categorical variables were computed using the chi-square test. Femoral head collapse was defined as the primary endpoint event, specifically referring to a loss of femoral head height of *>*2 mm on anteroposterior (AP) radiographs, compared with baseline images obtained after the initial reduction.

The distribution of repair band types differed significantly between the two groups. Specifically, the proportion of medial repair bands was markedly higher in the collapse group than in the non-collapse group (84.13% vs. 27.06%, *P* < 0.001). Furthermore, the proportion of completely displaced FNF was significantly greater in the collapse group (57.14% vs. 37.65%, *P* = 0.021). Survival analysis revealed that patients with a medial repair band had a significantly lower femoral head survival rate (*P* < 0.001). The median time to collapse was 27.00 months for medial repair bands vs. 29.50 months for lateral repair bands (Figure 4). The clinical outcomes of two representative cases with distinct fracture types are illustrated in Figure 2.

**Figure 4:**
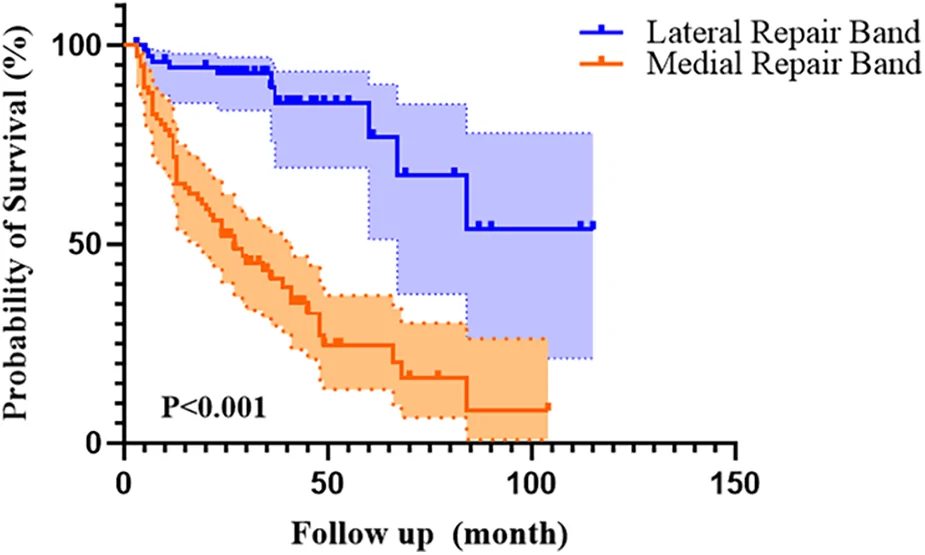
Survival analysis of femoral head collapse based on different repair band type.

Univariate logistic regression analysis demonstrated that complete fracture displacement (OR: 2.208, 95% CI: 1.136–4.292, *P* = 0.019) and the presence of a medial repair band (OR: 14.287, 95% CI: 6.242–32.699, *P* < 0.001) were potential risk factors for femoral head collapse. Based on the results of univariate analysis, this study incorporated variables with *P* < 0.05 (namely, complete fracture displacement and medial repair zone) into a multivariate logistic regression model for further analysis. The results confirmed that the medial repair band was an independent risk factor for collapse in TONFH (OR: 13.350, 95% CI: 5.767–30.907, *P* < 0.001) (Table 2). Figure 3 illustrates the outcomes of two representative cases with Garden type IV FNF.

**Table 2:** Univariate and multivariate logistic regression analyses of variables associated with progression to femoral head collapse.

Variable	Univariate analysis		Multivariate analysis	
	OR (95% CI)	*P*-value	OR (95% CI)	*P*-value
Medial Repair Band	14.287 (6.242–32.699)	<0.001	13.350 (5.767–30.907)	<0.001
Complete Displacement	2.208 (1.136–4.292)	0.019	1.398 (0.628–3.114)	0.412
Anatomical Reduction	0.606 (0.314–1.170)	0.136		
Surgery within 24h	0.901 (0.456–1.178)	0.918		
Sex	0.758 (1.002–1.040)	0.408		
Age	1.002 (0.966–1.040)	0.911		

Univariable analysis revealed that complete fracture displacement (Garden type IV) was significantly associated with the formation of a medial repair band (OR: 2.750, 95% CI: 1.409–5.368, *P* = 0.003), representing an approximately 2.75-fold increased risk compared with incompletely displaced fractures. Univariable analysis also showed no significant associations with age or time to anatomical reduction. In the multivariate analysis, besides including the variable with *P* < 0.05 from the univariate analysis (complete fracture displacement) into the model, we additionally forced the inclusion of reduction quality and timing of surgery as covariates for adjustment based on clinical significance. After adjusting for reduction quality and surgical timing, multivariable analysis confirmed that complete fracture displacement remained significantly associated with the formation of a medial repair band (OR = 2.581, 95% CI: 1.304–5.106, *P* = 0.006) (Table 3). This indicates that, irrespective of reduction quality or surgical timing, complete fracture displacement increases the risk of medial repair band formation by approximately 2.58-fold.

**Table 3:** Univariate and multivariate logistic regression analyses for the association between complete displacement and medial repair band formation.

Variable	Univariate analysis		Multivariate analysis	
	OR (95% CI)	*P*-value	OR (95% CI)	*P*-value
Complete Displacement	2.750 (1.409–5.368)	0.003	2.581 (1.304–5.106)	0.006
Surgery within 24h	1.753 (0.866–3.468)	0.107	1.203 (0.522–2.007)	0.947
Anatomical Reduction	0.937 (0.490–1.791)	0.844	1.479 (0.728–3.003)	0.279
Sex	1.656 (0.862–3.183)	0.130		
Age	1.032 (0.994–1.071)	0.104		

## Discussion

In this retrospective analysis of 148 patients with TONFH, the results demonstrated that the medial repair band was an independent risk factor for femoral head collapse. Furthermore, the study indicated that complete fracture displacement was significantly associated with the formation of a medial repair band. Using the fovea of the ligamentum teres as an anatomical landmark, evaluating the extent of the repair band on coronal MRI may provide a simple and reproducible method for assessing the risk of collapse in TONFH.

The formation of medial and lateral repair bands on MRI is pathologically rooted in the vascular distribution within the femoral head, particularly the perfusion territories of the superior retinacular artery (SRA) and inferior retinacular artery (IRA), along with their anastomotic network (22). The anastomotic site between the SRA and IRA lies adjacent to the fovea of the ligamentum teres (23). Recent studies have demonstrated that this arterial anastomotic network can provide compensatory perfusion in cases of localized femoral head ischemia (22, 24–28). In non-displaced or partially displaced fractures (Garden types I–III), although the SRA system may be compromised, the IRA system often maintains intact compensatory capacity. This results in limited necrotic areas, manifesting on coronal MRI as a repair band that terminates lateral to the fovea (Figure 2A). In contrast, in completely displaced fractures (Garden type IV), both the SRA and IRA systems are severely damaged, leading to extensive necrosis. On coronal MRI, this manifests as a repair band that extends medial to the fovea (Figure 2B). The findings of this study corroborate the aforementioned pathological mechanisms. Univariate analysis revealed a significant association between complete fracture displacement and the formation of a medial repair band, whereas multivariate analysis identified the medial repair band as an independent predictor of femoral head collapse. These results suggest that complete fracture displacement can cause extensive damage to the superior and inferior supporting arteries, and coupled with insufficient vascular recanalization in the femoral head following cannulated screw fixation, together lead to hemodynamic impairment and promote the extension of the repair band to the medial fovea capitis. The medial repair band then induces an imbalance in bone remodeling by obstructing blood flow, ultimately promoting femoral head collapse under weight-bearing forces.

Our study revealed that the medial repair band was present in 84.13% of patients in the collapse group. Therefore, extension of the repair band medial to the fovea of the ligamentum teres should be considered a key early warning sign of impending collapse in TONFH. In contrast, cases exhibiting only a lateral repair band were associated with favorable outcomes, suggesting preserved compensatory perfusion from the inferior retinacular artery and a robust reparative potential of the femoral head. Lee et al. (29) followed 33 cases of focal TONFH involving only the lateral area for 6 years, and none developed collapse, which is consistent with the outcomes of patients with a lateral repair band in our study. Boraiah et al. (27) quantitatively evaluated blood supply using gadolinium-enhanced MRI and found that the inferior retinacular artery can contribute up to 20% of the total perfusion of the femoral head, further confirming its critical role in maintaining femoral head vascularity. Zhao et al. (22) demonstrated through micro-CT and digital subtraction angiography (DSA) that the inferior retinacular artery system serves as an important structure preserving blood supply after FNF, and its compensatory capacity helps sustain perfusion. Thus, the use of the medial repair band as an imaging feature indicative of collapse risk in TONFH is well-supported by anatomical evidence.

The present study demonstrated that complete fracture displacement increases the risk of medial repair band formation by 2.58-fold. Zhao et al. (30) also revealed through angiographic studies that the degree of fracture displacement is significantly correlated with the severity of vascular injury—greater displacement leads to more severe vascular damage and a corresponding increase in the incidence of TONFH. These findings support the value of the Garden classification as an important tool for assessing femoral head stability and predicting collapse. Furthermore, some scholars (31) have suggested that Garden type IV fractures may contain subtypes, as outcomes still vary considerably within this group, as illustrated by the different clinical courses shown in Figure 3. Therefore, this study provides new imaging-based evidence for the potential subclassification of Garden type IV fractures.

Currently, classification methods for assessing ONFH have been progressively refined (32–35). However, their clinical application still faces significant challenges. Because people more hope to assess the risk of TONFH collapse and intervene before its occurrence. In this study, MRI was performed at 3 months after FNF surgery, and the ligamentum teres fovea was used as an anatomical landmark to differentiate between medial and lateral repair bands. Our results demonstrate that the medial repair band is an independent risk factor for collapse in TONFH. We propose that this imaging feature offers relatively high accuracy and good reproducibility for assessing the likelihood of femoral head collapse.

Limitations: Due to its retrospective design, this study inevitably had selection bias, loss to follow-up, and missing data, issues that can be effectively addressed in future prospective studies. As a single-center study with a limited total number of enrolled patients, a larger-scale, multicenter study with long-term follow-up is needed to further validate the results. At the statistical methods level, this study could only adjust for measurable confounding variables and was unable to control for unmeasured confounding factors, which may have somewhat affected the accuracy of the results. In terms of imaging evaluation, the failure to routinely include axial and sagittal MRI sequences may have limited the comprehensive assessment of the full extent of the repair band. Finally, since all MRI examinations were uniformly performed at 3 months postoperatively, only the morphology of the repair band at a single time point was evaluated, as the evolution of the repair band is a dynamic process, single time-point evaluation may not fully capture its dynamic characteristics, which to some extent limits the generalizability of the findings of this study.

In conclusion, we determined that the MRI medial repair band is an independent risk factor for femoral head collapse and is closely associated with complete fracture displacement. The MRI medial repair band, as an imaging feature for predicting collapse in TONFH, possesses accuracy and good reproducibility.

## Data Availability

The data analyzed in this study is subject to the following licenses/restrictions: To protect privacy and respect confidentiality, no raw data have been made available in any public repository. The datasets used and analyzed during the current study are available from the corresponding author on reasonable request. Requests to access these datasets should be directed to Zhikun Zhuang: qlflynn@163.com.
